# News and Coming Events

**Published:** 1909-04-24

**Authors:** 


### NEWS AND COMING EVENTS.

The Festival Dinner of the City of London Lying-in Hospital will be held at the Trocadero Restaurant on Thursday, April 29, at 7 for 7.30 p.m., when the Hon. Rupert Guinness, C.M.G., M.P., will be in the chair.

The Festival Dinner of the Royal Hospital for Incurables, Putney Heath, will be held on Wednesday, May 5, at the Savoy Hotel, Strand, when the Lord Mayor will be in the chair. The dinner will be at 6.30 for 7 p.m. precisely.

A Festival Dinner in aid of the East London Hospital for Children, Shadwell, E., will be held at the Ritz Hotel on Wednesday, April 28, at 7.30 p.m., when the chair will be taken by Field Marshal Lord Grenfell, P.C., G.C.B., G.C., M.G.

Lord George Hamilton, speaking on April 5 at the annual meeting of the Victoria Hospital, Deal, of which he is president, said the Poor Law Commission looked very closely into the question of medical relief for the wage-earning classes. Putting aside as impracticable a scheme of universal gratuitous medical relief, which would probably cost thirteen millions a year and possessed other disadvantages, and rejecting the German system of compulsory insurance, they came to the conclusion that there should be a general system of medical relief on a provident basis, and that those who had made provision against illness should in the event of their requiring hospital treatment be entitled to gratuitous relief on production of a medical certificate. They were in favour of trusting to persuasion and inducement to utilise as far as possible the existing provident dispensaries all over the country as a basis on which they could if necessary extend the system of medical relief.

The annual general meeting of Governors of the Royal Eye Hospital, St. George's Circus, Southwark, S.E., will be held at the hospital on Wednesday, April 28, at 4 p.m.

The annual report of St. Thomas's Hospital shows that last year 561 beds were available for use, and the average number occupied daily was 498. The number of in-patients was 6,972 and the average length of their stay was 26 days. The total cost of each in-patient was £7 11s. 6d. New out-patients numbered 82,569, with 226,239 attendances. Seventy-eight inquests were held during the year. In the *x*-ray department 3,477 cases were treated and 5,086 skiagrams were taken. It is proposed to open two of the three empty wards with as little delay as possible—one for female medical cases and the other as a maternity ward for special cases. The ordinary expediture last year was £64,278. Legacies produced £17,341, and the total income was £76,126.

The Post-graduate College of Medicine and Surgery attached to the West London hospital has had a satisfactory year of work, according to the annual report of the hospital. Two hundred and thirty-two fresh names have been enrolled on its books, and many old members have returned for a further period of study. The total number of medical men who have attended the college now amounts to 1,654, and includes 67 life members. Amongst those attending last year were 24 officers in the Naval Medical Service, 26 in the Royal Army Medical Corps and Indian Medical Service, and 48 practitioners from the Colonies and abroad. The West London Medico-Chirurgical Society, which holds its meetings in the rooms of the Post-Graduate College, is also in a flourishing condition. At the date of the last report it had upon its books 641 members.

The Lord Mayor will preside at a festival dinner in aid of the funds of the City of London Hospital for Diseases of the Chest, Victoria Park, to be held at the Trocadero Restaurant on May 6, at 7.30 p.m.

A Government grant of £200 has recently been made to Mr. Harry W. Cox, a manufacturer of *x*-ray apparatus and other scientific instruments, on account of his sufferings through the use, partly of an experimental nature, of Röntgen-ray apparatus.

A serious epidemic of typhoid fever has for some time been raging at Cherbourg. The troops in particular have suffered severely from the disease, and 190 cases are still under treatment in the military hospital. The outbreak is stated by the *Times* Paris correspondent to be due to the unsatisfactory character of the water supply and to the primitive sanitary arrangements.

According to an official return the total number of deaths from industrial accidents reported in 1908 was 4,224, a decrease of 253 on 1907, but an increase of 29 on the mean of the five years 1904-1908. A decrease is recorded in every group of occupations except mining and quarrying.

Princess Alexander of Teck opened on April 20 the first block of the new buildings of the Royal National Orthopædic Hospital in Great Portland Street, comprising the out-patient department and nurses' home, and including a new orthopædic gymnasium, as well as massage and electrical departments. The new buildings have cost some £75,000, of which £25,000 is still to be raised. The hospital itself is to be opened during the summer.

Sir William Clerke presided at the annual meeting of the Hospital for Women, Soho Square. During 1908 the number of in-patients treated was 946, which is greater than in any previous year. There were 3,810 out-patients, with a total of 14,715 attendances. The committee of management appeal for increased financial support for maintenance and also towards the reduction of a mortgage debt of £7,000. The work of rebuilding the hospital has been commenced, and by the end of the present year it is hoped that a practically new hospital will be ready for the reception of patients. The entire scheme will cost £18,500, and towards this sum the committee had in hand or promised £10,500, and an additional £3,000 had been promised by King Edward's Hospital Fund for London, on condition that the balance required to complete the work—namely £5,000—was raised by next October.

